# The challenges of single cell transcriptomics on difficult human tissue: the placenta

**DOI:** 10.1093/nargab/lqag079

**Published:** 2026-07-16

**Authors:** Theodoros Xenakis, George T Hall, Jose J Moreno-Villena, Sara L Hillman, Yara E Sanchez-Corrales, Sergi Castellano

**Affiliations:** Genetics and Genomic Medicine Research & Teaching Department, UCL Great Ormond Street Institute of Child Health, London WC1N 1DZ, United Kingdom; Genetics and Genomic Medicine Research & Teaching Department, UCL Great Ormond Street Institute of Child Health, London WC1N 1DZ, United Kingdom; Genetics and Genomic Medicine Research & Teaching Department, UCL Great Ormond Street Institute of Child Health, London WC1N 1DZ, United Kingdom; Fetal Medicine Unit, University College London Hospitals (UCLH) NHS Foundation Trust, London NW1 2BU, United Kingdom; Institute for Women’s Health, University College London (UCL), London WC1E 6DE, United Kingdom; Genetics and Genomic Medicine Research & Teaching Department, UCL Great Ormond Street Institute of Child Health, London WC1N 1DZ, United Kingdom; Genetics and Genomic Medicine Research & Teaching Department, UCL Great Ormond Street Institute of Child Health, London WC1N 1DZ, United Kingdom; UCL Genomics, UCL Great Ormond Street Institute of Child Health, London WC1N 1DZ, United Kingdom

## Abstract

Demand for the application of single cell transcriptomics on difficult tissues, processed and stored in disparate conditions, has led to the development of various single cell modalities. We focus on the placenta, a challenging tissue to transcriptomically interrogate due to senescence, intermittent hypoxia, high levels of RNase activity and tissue trauma at delivery. We performed single cell and nuclei transcriptomics on two samples with probe-based and native molecule transcript capture, droplet-based and *in situ* plate-based cell isolation. We explored sample and storage variations, including freshly dissociated tissues, fixed cells, snap frozen, and formalin fixed paraffin embedded processing. We find that variations in sample processing and storage have a much larger effect on cell-type proportions than differences in chemistry, impacting the biology that can be learned or the identification of disease markers. Further, the transcriptomic output of *in situ* combinatorial indexing consistently overlaps with single-nucleus transcriptomics, sharing little with other modalities. This may limit combinatorial indexing for sampling cytoplasmic messenger RNAs. Our comprehensive analysis of the technical variance between single cell transcriptomic modalities, including their sample management strategies, provides novel and essential considerations for the experimental design and analysis of single cell transcriptomics applicable to challenging tissues, offering actionable guidance for future experiments.

## Graphical Abstract

**Graphical Abstract:**
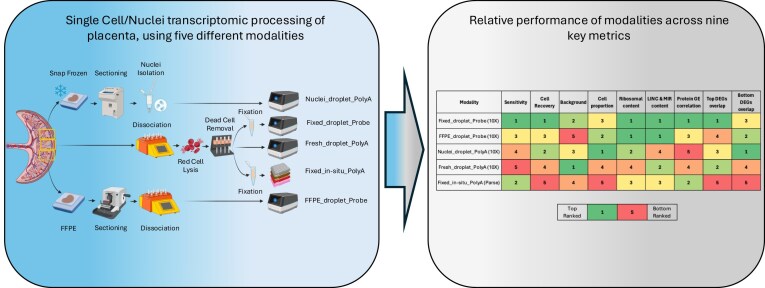

## Introduction

Biological tissues are inherently complex and heterogenous. They comprise cell types and sub-types that can vary in their individual and functional states according to both external factors, such as cell signalling from other cells, and internal processes, such as cell cycle phase or apoptosis. This variation is exacerbated when tissues are affected by pathological changes [1]. Single cell transcriptomics quantifies this cellular heterogeneity, in health and disease, with promising applications in basic and translational research [2].

The popularity of single cell transcriptomics has justifiably increased, with rising demand for its application on increasingly difficult tissues with varying processing and storage conditions. Previously, freshly dissociated viable cells were the only option as an input in most commercially available solutions, limiting the application of single cell transcriptomics. In recent years, methodological modalities have been developed to enable single cell transcriptomic analysis of isolated nuclei from snap frozen tissue, cells fixed after tissue dissociation and cells isolated from formalin fixed paraffin embedded (FFPE) tissue blocks. This was enabled by optimising transcript capture using probe sets that target protein-coding genes, as opposed to oligonucleotides that capture the 3′ prime poly-adenylated tails of messenger RNA (mRNA) molecules. Furthermore, novel plate-based methods promise the opportunity to circumvent cell-shape capture limitations inherent to droplet-based methods.

Previous comparative studies have mostly interrogated either naïve or sorted peripheral blood mononuclear cells (PBMCs) and cell lines. They have demonstrated the variance in performance and functional outputs using different commercially available kits, exploring their effects on sample multiplexing, case control comparisons, and drug screening potential [3–10]. In an analogous fashion, commercially available solutions have been developed and optimized on similar common sample types such as PBMCs and then retrospectively validated on other sample types.

Here, we focus on a more challenging tissue, the placenta, allowing us to better understand the limitations of different modalities in less optimal sample types. The placenta is the site of direct molecular and cellular communication between the pregnant woman and the fetus and, has traditionally been considered a difficult tissue to transcriptionally interrogate, particularly from pregnancies affected by pathology. This is due to senescence, intermittent hypoxia, high levels of RNase activity and tissue trauma at delivery [11, 12]. Understanding the effect of these issues may be broadly applicable as similar difficulties have been reported on pancreas and liver [13, 14]. These tissues, among others, pose a challenge for transcriptomic solutions that rely on capturing native mRNA molecules from their 3′ prime, poly-adenylated tail (polyA), which are sensitive to degradation. A challenge that may be overcome using near whole transcriptome probe sets.

Furthermore, early studies on single cell methodologies on the placenta have predominantly focused on differences between single nuclei and single cell methods, where important findings regarding cell-type resolution and loss of disease signatures have been reported [15, 16]. However, appropriate representation of certain cell types in the placenta, such as the multinucleated syncitiotrophoblasts (STBs) [17], remains challenging with droplet-based modalities due to cell shape and size restrictions [18]. These challenges have also affected the interrogation of binucleated cardiomyocytes in heart tissue and neurons in brain tissue, further highlighting the importance of understanding how different modalities can overcome these challenges [19, 20].

To understand and quantify the technical variance brought by the different single cell transcriptomic modalities on this difficult tissue, including different sample storage and preprocessing, we performed single cell and nuclei transcriptomics on two donors (biological replicates) using five different modalities. We report that sample storage and processing have significant impact on cell-type population ratios. Furthermore, we present evidence that *in situ* combinatorial indexing consistently shares similarities in transcriptomic output with single nuclei transcriptomics and yet shows distinct lack of overlap with other modalities of the most overexpressed genes. Although new versions of kits and updated chemistries may improve some of these limitations across modalities, our findings fundamentally relate to their underlying mechanisms, which, in conjunction with the impact from processing and storing samples, are likely intrinsic to each single cell technology. Finally, we offer guidance on the design of single cell experiments for each modality in a simple way (Fig. 5c).

## Materials and methods

### Patient recruitment

We recruited pregnant women at the fetal medicine unit, antenatal care unit and labour ward of the University College London Hospitals, NHS Foundation Trust. The study cohort comprised three pregnant women. The study was approved by the South Oxford Research Ethics Committee (REC CODE: 17/SC/0432).

Inclusion criteria were women with a singleton pregnancy, able to consent, experiencing preterm delivery by C-section due to iatrogenic reasons prior to 37 weeks of gestation. Exclusion criteria were women with pregnancies affected by major fetal anomalies (whether chromosomal, structural, or genetic), twin pregnancies, and comorbidities including diabetes mellitus (gestational or T1 or T2), maternal autoimmune, renal or heart disease. Donors were excluded if they had any clinical symptoms/signs of infection or a positive test of infection during standard clinical practice.

Placentas from two of the recruited patients were used for the single cell/nuclei modality comparison and the placenta of the remaining patient was used for i*n situ* spatial transcriptomics, relevant to testing cell-type proportions.

### Sampling

After delivery, the placentas were washed twice in 1× DPBS (-Ca, -Mg). The largest distance between the cord insertion site and the edge of the placenta was then established. From the mid-point of said distance three adjacent 2 cm × 2 cm samples were taken. One was placed in cold HypoThermosol FRS (Sigma–Aldrich, cat no: H4416) at 4°C for a maximum of 24 h, for dissociation and single cell sequencing. The second was snap frozen in an isopentane bath set in dry ice and embedded in OCT, and stored at −80°C. The third was placed in formalin saline solution for paraffin embedding to prepare FFPE blocks. All sampling occurred within 30 min of delivery to ensure RNA integrity.

### Dissociation

Samples were removed from HypoThermosol FRS and transferred to C-Tubes (Milteny Biotec, cat no: 130-093-237) on ice before being minced with scissors. Ten milliliters of Accutase (Sigma–Aldrich, cat no: A6964) was then added to the C-Tubes. The tissues were then dissociated on a gentleMACS Octo dissociator using a custom protocol. Dissociated cell suspensions were then passed through stacked 100 μm and 70 μm cell strainers (Milteny Biotec, cat no: 130-098-463, 130-098-462), pre-wetted with 2 ml of Dulbecco’s modified Eagle’s medium (DMEM), 10% fetal bovine serum (FBS). Cell strainers were then rinsed with a further 10 ml of DMEM, 10% FBS.

Cells were pelleted by centrifugation at 300RCF for 5 min. The supernatant was then removed, and the pellets were resuspended in 20 ml of Red Cell Lysis buffer (Myltenyi Biotec, cat no: 130-094-183) and incubated for 10 min at room temperature on a rocker. Then 30 ml of cold 1× DPBS was added, and cells were pelleted by centrifugation at 300 RCF for 5 min. The supernatant was removed, and cells were washed by resuspending in 5 ml DMEM, 10% FBS before being pelleted by centrifugation at 300 RCF for 5 min.

The supernatant was then removed, and cell pellets were resuspended in 1 ml of Dead Cell Removal MicroBeads (Miltenyi Biotec, cat no:130-090-101) and incubated for 15 min at room temperature. Suspensions were then loaded onto MACS LS columns (Miltenyi Biotec, cat no:130-042-401) pre-rinsed with 3 ml 1× Binding buffer. Columns were rinsed with a further 3 ml of Dead Cell Removal 1× Binding buffer. Cells were then pelleted by centrifugation at 300 RCF for 5 min. The supernatant was removed, and cells were resuspended in 1 ml DMEM, 10% FBS.

Final cell suspensions for placental samples of both patients were divided into three. One to be used immediately with the 10X Genomics Next GEM Single cell 5′ v2 kit (Fresh_droplet_Poly-A). The second was fixed according to the Parse Biosciences Evercode Cell Fixation v2 User Manual v2.3 to be used with the Parse Biosciences Evercode WT Mini v2 kit (Fixed_in-situ_Poly-A). The third was fixed according to 10X Genomics demonstrated protocol CG000478 rev D to be used with the 10× Genomics Chromium Fixed RNA Profiling Reagent Kit (Fixed_droplet_Probe).

### Nuclei isolations from placenta samples

Snap frozen, OCT embedded placental samples were cryosectioned on a Bright OTF5000 cryostat. Five 25 μm sections were collected. Nuclei were isolated from the sections, using Chromium Nuclei Isolation Kit (10X Genomics, cat no: 1000494), following 10X Genomics user guide CG000505, to be used with 10X Genomics Next GEM Single cell 5′ v2 kit (Nuclei_droplet_Poly-A).

### FFPE dissociations from placental samples

FFPE blocks from placenta samples of both patients were sectioned on a microtome. Five 25-μm sections were collected and dissociated according to 10X Genomics demonstrated protocol CG000632 revD to be used with the 10X Genomics Chromium Fixed RNA Profiling Reagent Kit (FFPE_droplet_Probe).

### Generating single cell transcriptomic libraries

Dissociated cells and isolated nuclei were checked for concentration and viability using an Acridine Orange/Propidium Iodide Stain (logos Biosystems, cat no: F23001) on a logos Biosystems Luna FL automated cell counter. Cells/nuclei for each sample were used to generate single cell/nuclei transcriptomic libraries according to the specifications listed in Table 1.

**Table 1.** Library preparation details per single cell modality

Modality	Cell origin	Single cell sequencing kit	Library prep user guide followed
Fresh_droplet_PolyA	Cells dissociated from fresh tissue	Chromium Next GEM Single cell 5′ Kit v2 kit	10X Genomics CG000331
Nuclei_droplet_PolyA	Nuclei from snap frozen tissue	Chromium Next GEM Single cell 5′ Kit v2 kit	10X Genomics CG000331
FFPE_droplet_Probe	Cells dissociated from FFPE blocks	Chromium Fixed RNA Profiling Reagent Kit	10X Genomics CG000691
Fixed_droplet_Probe	Cells dissociated from fresh tissue then fixed	Chromium Fixed RNA Profiling Reagent Kit	10X Genomics CG000691
Fixed_in-situ_PolyA	Cells dissociated from fresh tissue then fixed	Evercode WT Mini v2 kit	Parse Biosciences Evercode WT Mini v2 User Manual v2.3.

### Sequencing

Resulting single cell and single nuclei transcriptomic libraries were sequenced on an Illumina Novaseq 6000 sequencer using S4 (200 cycle) v1.5 sequencing kits (Illumina, cat no: 20028313). Libraries were sequenced over the minimum recommended depth per library type (Supplementary Table S2).

### *In situ* spatial transcriptomics

An 8-μm section of placenta was loaded onto specialized slide to be processed on the 10X Genomics Xenium Analyser (10X Genomics, cat no’s: 1000460, 1000487, 1000461) using the Xenium Human Multi-Tissue and Cancer Panel (10X Genomics, cat no: 1000626) and a Xenium Add-on Custom 51 to 100 Gene Panel (10X Genomics, cat no: 1000651) for a total of 477 genes, following 10X Genomics demonstrated protocol CG000581 and user guides CG000582 and CG000584. The gene selection for the Add-on Custom 51 to 100 Gene Panel was based on differentially expressed genes (DEGs) in STB and cytotrophoblast (CTB) from a previous study [21].

Pre-processed data files generated from the Xenium instrument on-board analysis software were used to generate a Seurat object using Seurat. We filtered out cells with zero transcript counts and normalized the data (‘SCTransform’). We then performed principal component analysis, k-nearest-neighbour calculation (‘FindNeighbors’) and graph-based community detection using Louvain clustering (‘FindClusters’). We annotated cell types using gene expression of manually curated genes from the literature (Supplementary Fig. S3 and Supplementary Table S1). Cell types were annotated at two levels of resolution. First, simply into immune and non-immune groups. Subsetting each group and repeating the clustering pipeline allowed for another level of annotation with higher granularity. During clustering, we detected cell with mixed profiles of mutually exclusive marker genes for different cell types, which were also excluded.

### Count matrix generation and background removal

As each modality has fundamentally different mechanisms of transcript capture and/or transcript availability (e.g. nuclear transcripts versus whole cell transcripts versus transcripts only present in a probe set), joint data clustering and cell-type annotation could be adversely affected by technical variance and therefore impact our ability to interrogate it. As such, we only integrate libraries within the same modality or libraries for probe-based modalities prior to annotation, as the mechanisms of transcript capture and/or transcript availability are the same for these. We then applied the same analytical strategy across modalities as described below.

#### Droplet based

For each single cell and single-nucleus library, the raw bcl files were converted into fastq files using cellranger ‘mkfastq’. Reads were aligned to the GRCh38-2020-A human reference genome (GENCODE v32/Ensembl98 distributed by 10X Genomics) and a matrix of unique transcripts per cell for each library was obtained using cellranger ‘multi’.  We used CellBender 0.2.0 [22]. to correct for ambient RNA per library and classify cell-containing droplets from empty ones.

#### *In situ* combinatorial indexing

For each single cell library, the raw bcl files were converted into fastq files using bcl2fatsq. To minimize bias in gene detection and ensure the fidelity of the comparisons across all single cell transcriptomic modalities, reads were aligned again to the GRCh38-2020-A human reference genome (GENCODE v32/Ensembl98 distributed by 10X Genomics) and a matrix of unique transcripts per cell for each library was obtained using split-pipe ‘all’ and ‘comb’.  In order to appropriately calculate ambient RNA, using Seurat 5.1.0 [23] we merged the unfiltered library matrixes and generated a .h5ad object. We then used CellBender 0.2.0 to correct for ambient RNA.

### Quality control, doublet removal, and cell type annotations

We filtered low quality cells using Seurat 5.1.0. We excluded cells with number of detected genes below 400 and above 6000, with transcript counts above 17 500, with percentage of mitochondrial reads more than 10% and percentage of haemoglobin genes more than 0.25%.

Doublets where computationally assessed and removed using DoubletFinder [24] assuming 0.75% doublets per 1000 cells. Notably, following a less stringent approach on doublet removal by solely relying on upper thresholding of unique molecular identifiers (UMIs) per cell, alongside removal of cells with mutually exclusive gene expression profiles did not change our results. This approach could be used when maximally maintaining cell numbers is of paramount importance in a challenging tissue to sample. Nevertheless, here we present results following the most conservative doublet removal by combining both approaches.

Data was normalized (‘LogNormalise’) and scaled (‘ScaleData’) for single cell/nuclei libraries. Highly variable genes selection was used to perform principal component analysis, k-nearest-neighbour calculation (‘FindNeighbors’) and graph-based community detection using Louvain clustering (‘FindClusters’).

We annotated cell types using gene expression of manually curated genes from the literature (Supplementary Fig. S3 and Supplementary Table S1). Cell types were annotated at two levels of resolution. First, simply into immune and non-immune groups. Subsetting each group and repeating the clustering pipeline allowed for another level of annotation with higher granularity. During clustering, we detected additional doublet clusters (identified by a mixed profile of mutually exclusive marker genes for different cell types), which we also excluded.

### Cell-type proportions, gene expression metrics, and cell capture rates

The aim of this manuscript is to quantify and present the technical variance between different modalities. To present the effect of this technical variance, we generated UMAPs using Harmony [25] v1.2.0 (Supplementary Fig. S7) alongside UMAPs without Harmony applied, illustrating the effect of the technical variance, while also providing reassurance of the accuracy of our cell-type anotation. All downstream analysis were therefore performed on data integrated in a manner that preserves the technical variance between modalities in order for it to be quantifies and explored.

Filtered and annotated libraries were merged, normalized (‘LogNormalise’) and scaled (‘ScaleData’) using Seurat 5.1.0, and cell-type annotations were checked for consistency. Cell-type proportions for each method where then calculated for both levels of annotations.

We have furthermore statistically assessed differences in cell-type proportions. For each sample (modality or library), we calculate the sum of absolute differences between its proportions of cell types and those in the *in situ* spatial transcriptomics sample, as we previously measured in [26]. We derived a 95% confidence interval on this statistic (z, defined below) using bootstrap resampling with 10 000 resamples of cells/nuclei for each sample (Supplementary Fig. S5). This statistical assessment also supports our claims, again when examining our results per modality and per donor.

To define the statistic in more detail, let the vector *x* contain the proportion of each cell type in sample *X*, i.e. ${{x}_i}$ is the proportion of cells/spots in sample *X* labelled as cell-type *i*. Define *y* similarly for sample *Y*. We use bootstrap resampling to derive confidence intervals on the sum of the absolute differences of the proportions of each cell type between *X* and *Y*:


\begin{eqnarray*}
z\ = \ \mathop \sum \limits_i \left| {{{x}_i}\ - \ {{y}_i}} \right|.
\end{eqnarray*}

Percentages of gene expression per cell for three different classes of genes were calculated for each modality. These were long intergenic non-coding (LINC), ribosomal protein (RP), and micro RNA (MIR) genes. The number of cells remaining post quality control and doublet filtration was then calculated as a percentage of the number of cells inputted during sample processing.

### Differential abundance between biological replicates and across modalities

We ran Dawnn (v1.1.0) [27] on each pair of libraries, searching for absolute differential abundance and using other default settings. For each pair of libraries, we created a subset containing only cells/nuclei from these libraries and processed it using the standard normalization and scaling pipeline. We then ran Dawnn on the resulting PCA reduction.

### Computational downsampling of sequencing reads

We used the computational downsampling performed by Cellranger and split pipe to calculate the genes per cell in relation to reads per cell for each library as a whole.

We then exported the cell barcodes for cells we annotated as STBs and NK/Ts separately and per modality using Seurat. Using the 10X Genomics subset-bam v1.1.0 tool, we subsetted the .bam alignment files, generated by Cellranger and split pipe during count matrix generation, based on the exported cell barcodes. The resulting .bam files were the sorted and indexed using samtools ‘sort’ and ‘index’. Total reads in each new .bam file was established using samtools ‘view -c’.

Using UMI-tools v1.1.2, we sequentially downsampled each subsetted .bam file to a ratio of 0.05, 0.1, 0.2, 0.3, 0.5, 0.75, and 1.0 of its reads and extracted the number of UMIs at each downsampled depth and divided this number by the number of cells to calculate the number of UMIs per cell at each sequencing depth.

### Principal component analysis

The most consistently represented cell type across all modalities were the STB cells. As such using Seurat, we subsetted them and their gene expression matrixes to only include the genes that were shared between the probe set and poly-A capture approaches. After pseudobulking (‘AggregateExpression’) their gene expression by sample, we ran Principal Component Analysis (‘RunPCA’) using the top 2000 most variable genes per library with npcs = 8 and visualized the top 5 principal components in a UMAP plot (‘RunUMAP’).

We calculated the Euclidian distance in PC1 between each library using ‘dist’ (Base R) and averaged the distances by sample, excluding its biological replicate and by modality (Supplementary Fig. S8).

### Pseudobulking and gene expression correlation

The most represented cell type amongst the immune cell populations across the five modalities were the NK/T cells. Conversely from the non-immune population, as mentioned previously, the most represented cell type were the STB cells. Using Seurat, we subsetted these cell types, and gene expression matrixes were subsetted to only include the genes that were shared between the probe set and poly-A capture approaches. We then pseudobulked (‘AggregateExpression’), per cell type and modality, generating a per cell-type gene expression matrix for each modality. A correlation matrix was then calculated from the normalized transcript counts.

### Differential gene expression across modalities

In order to compare the number of DEGs across modalities, we again seperately subsetted the STBs and NK/Ts, performed pairwise DEG analysis using Wilcoxon rank-sum test analysis (Seurat function ‘FindMarkers’) and filtered the results for adjusted *P*-value below .05 and log_2_-fold change above 1 or below −1.

To ensure the key findings are confirmed using a pseudobulking approach, we repeated the DEG analysis using DESeq2 [28] v1.44.0 after pseudobulking (‘AggregateExpression’) the STBs and NKTs separately, per library (Supplementary Fig. S9).

### Inter-modality differential gene expression

To assess the overlap of DEG analysis outputs, we again subsetted the STBs. We performed pairwise Wilcoxon rank-sum test analysis (Seurat function ‘FindMarkers’) between the two biological replicates within each modality, retaining only DEGs with an adjusted *P*-value below .05 and ranked them by descending log_2_-fold change. We subsequently calculated the ratio of overlap for the top (most overexpressed) 10, 25, 50, and 100 DEGs independently as well as the bottom (most underexpressed) 10, 25, 50, and 100 DEGs.

### Comparative ranking of modalities

We summarize our results and rank each modality across the metrics presented in our results in the following manner:

Sensitivity: based on rate of increasing genes per cell as the reads per cell increase on the libraries as a whole, as well as based on rate of increasing UMIs per cell as the reads per cell increase for the subsetted STB and NK/T cells. The modality with the highest rate is ranked highest (ranked position: 1).
Cell Recovery: based on the percentage of cells loaded during processing, which remain in the final, filtered dataset. The modality with the highest percentage is ranked highest (ranked position: 1).
Background: based on the fraction of counts removed from cells by the background removal tool (CellBender). The modality with the lowest fraction is ranked highest (ranked position: 1).
Cell Proportions: based on the minimal observed compositional difference between each modality and *in situ* spatial transcriptomics, based on our bootstrapping analysis. The modality with the smallest observed compositional difference is ranked highest (ranked position: 1).
Ribosomal Content: based on the mean percentage expression of ribosomal genes in cells. The modality with the lowest mean is ranked highest (ranked position: 1).
LINC and MiR Content: based on the mean percentage expression of LINC and MIR genes in cells. The modality with the lowest mean is ranked highest (ranked position: 1).
Protein GE Correlation: based on correlation of pseudobulked protein coding gene expression of STB and NK/T cells between modalities. Correlation is first averaged between the two cell types and then across modalities. The modality with the highest average correlation across modalities is ranked highest (ranked position: 1).
Top DEG Overlap: based on the combined average of percentage overlap, of 10, 25, 50, and 100 most over-expressed genes, from differential gene expression analysis between biological replicates. Thus, considering not only the 100 most over-genes but the comparative ranking of these genes. The modality with the highest average overlap across modalities is ranked highest (ranked position: 1).
Bottom DEG Overlap: based on the combined average of percentage overlap, of 10, 25, 50, and 100 most under-expressed genes, from differential gene expression analysis between biological replicates. Thus considering not only the 100 most under-expressed genes but the comparative ranking of these genes. The modality with the highest average overlap across modalities is ranked highest (ranked position: 1).

## Results

### Assessment of single cell transcriptomic modalities in two biological replicates

**Figure 1.**
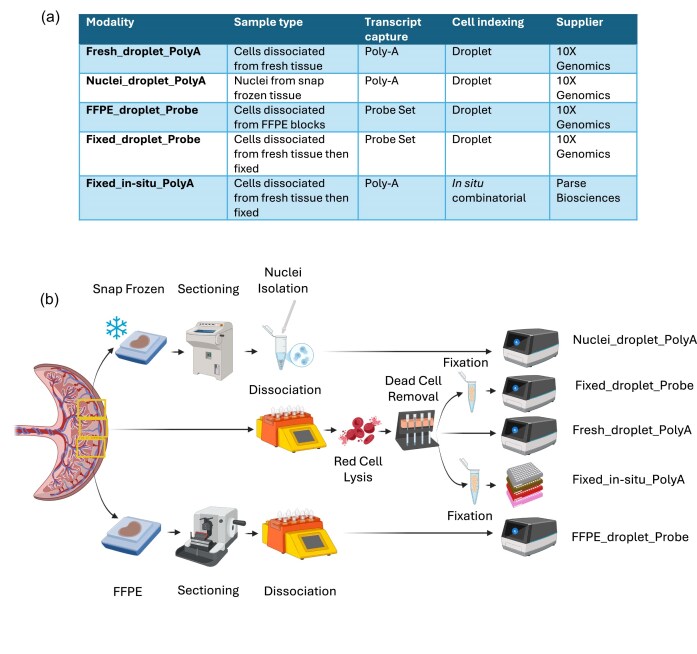
Overview of single cell/ nuclei modalities. (**a**) Table detailing the variable elements of the five different modalities interrogated. (**b**) Graphical representation of sampling and key processing steps for each modality.

To examine the technical variance between five different single cell modalities on the transcriptome, we processed two placental samples, which included the maternal decidua (Fig. 1a and b). These two samples were from late preterm deliveries, delivered at 37 and 36 gestational weeks, respectively.

**Figure 2.**
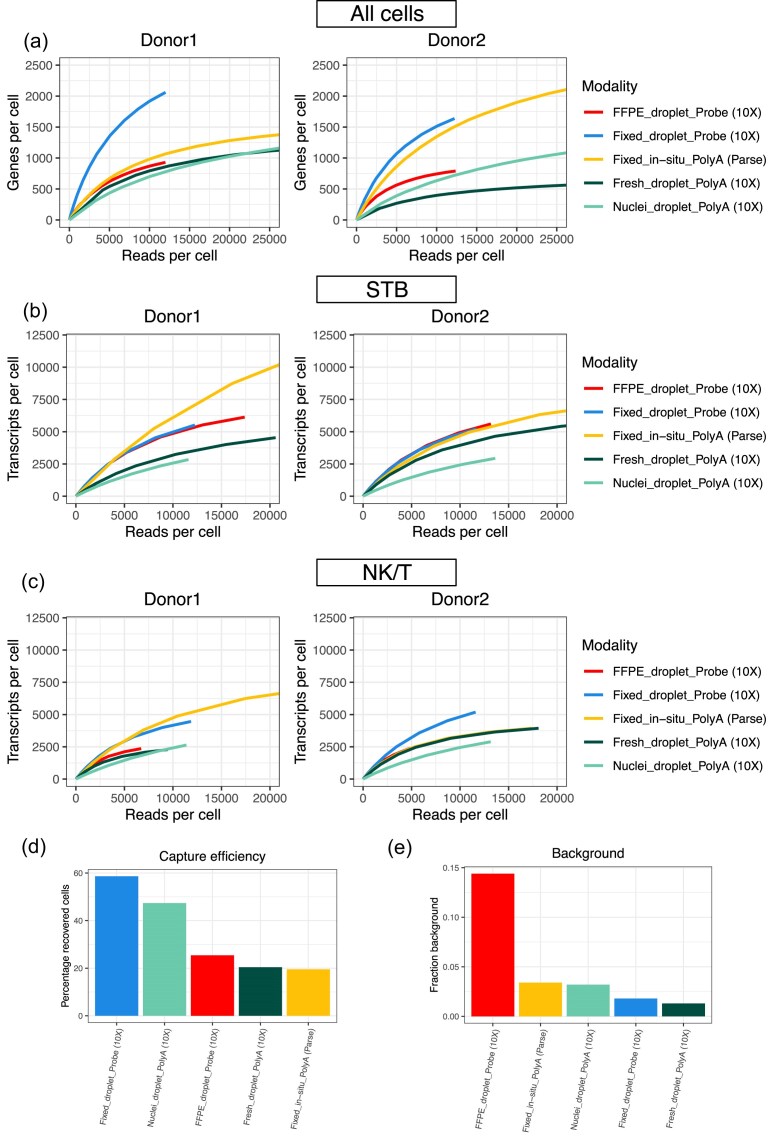
Performance metrics of modalities. (**a**) Sensitivity, as illustrated for each donor using the relationship between median genes per cell and median reads per cell for the five modalities (sequencing depth shown for each modality is to the manufacturers minimum recommendation +5%, Supplementary Fig. S1 presents the two donors together for each modality seperately). (**b**) Sensitivity for STBs, as illustrated for each donor using the relationship between average transcripts per cell and reads per cell for the five modalities. (**c**) Sensitivity for Natural Killer/T cells (NK/T), as illustrated for each donor using the relationship between average transcripts per cell and reads per cell for the five modalities. (**d**) Capture efficiency, showing the percentage of cells used for analysis post Quality Control filtering in relation to cells inputted for each modality. (**e**) Background, illustrating per modality the fraction of reads removed by the computational tool used for removing reads assigned to ambient RNA.

We appreciate that the low sample number in this study is a limitation, however, we consistently report low variance between the two samples in our analyses (Figs 2a–c, 4a and b, and Supplementary Figs S1, S2, and S4–S6).

### Chemistry-specific metrics

In the first instance, we examined the sensitivity of each modality by looking at the genes per cell in relation to the sequencing reads per cell (Fig. 2a and Supplementary Fig. S1). In both samples, the highest sensitivity was observed in Fixed_droplet_Probe, perhaps unsurprisingly given the probe-based transcript capture. The second most sensitive modality is Fixed_in-situ_PolyA, meaning the top two modalities utilize cells fixed after dissociation of fresh tissue.

As variance in cell-type composition as well as transcriptional diversity of different cell types can impact these sensitivity metrics, we looked at the transcripts (UMIs) per cell in relation to reads per cell for STB and NK/T cells separately for both donors (Fig. 2b and c), as the two most represented cell types. Fixed_droplet_Probe and Fixed_in-situ_PolyA remain the most sensitive methods at this resolution with Nuclei_droplet_PolyA showing the least sensitivity, perhaps unsurprisingly, given the absence of cytoplasmic RNA. Though taken together STB and NK/T cells represent approximately 65% of the cells analysed (Supplementary Table 3), the sensitivity of other cell types, present in too few numbers for us to explore, could differ.

To understand the cell capture efficiency of each modality, we quantified the percentage of cells remaining (from the total number of cells used as an input) after doublet and ambient RNA removal (background) and quality filtering (Fig. 2d). The highest cell capture efficiency was observed again in Fixed_droplet_Probe (57%) followed by Nuclei_droplet_PolyA (43%), where the capture efficiency is nearly triple and double, respectively, of the remaining three modalities. To understand the level of transcriptomic background introduced, we extracted the ‘fraction counts removed from cells’ metric outputted from the computational background removal tool used (Fig. 2e). The highest background was observed in FFPE_droplet_Probe (0.144%). This is in line with our expectations due to the challenging dissociation protocol required and the degraded nature of RNA from cells in FFPE tissue blocks. We observed good concordance between samples in both cell capture efficiency and background (Supplementary Fig. S2). These results offer important information for the consideration of experimental designs.

### Proportions of cell types

**Figure 3.**
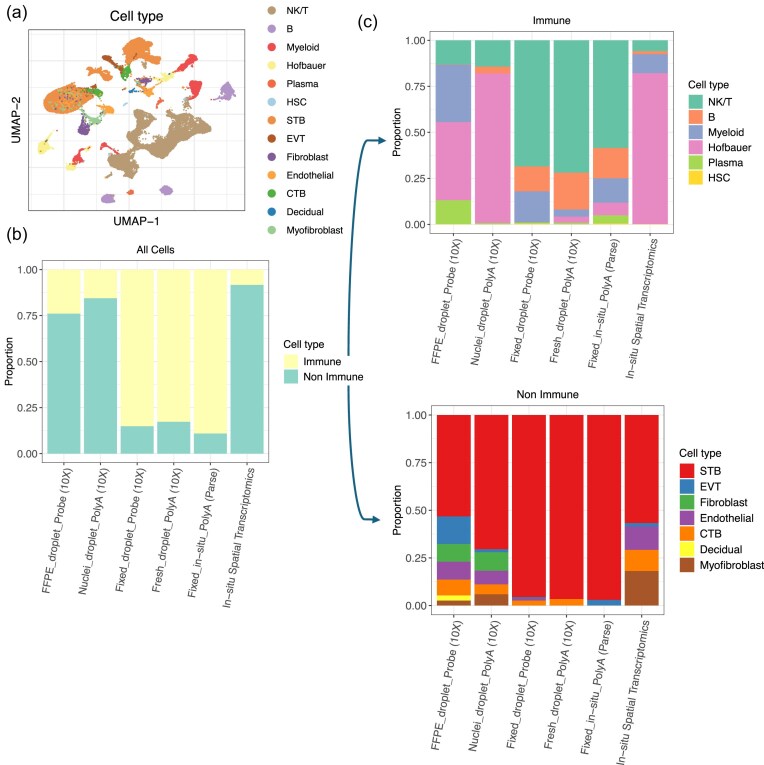
Cell-type proportions. (**a**) UMAP of combined dataset including all five modalities coloured by annotated cell type. (**b**) Relative proportion of cells annotated as immune and non-immune cells by modality. (**c**) Relative proportions of cell annotated within the immune and non-immune cell types.

After annotating the cell types represented in our final dataset (Fig. 3a and Supplementary Fig. S3a), we explored the proportion of each cell type per modality. We included carefully annotated *in situ* spatial transcriptomics from a placental sample of similar gestational age (35 weeks), to compare against a sample whose *in vivo* cell-type proportions have been maximally maintained, due to the maintenance of tissue architecture *in situ* Spatial Transcriptomics (Supplementary Fig. S3b and c). Initially, we annotated cells into immune and non-immune cell types (Fig. 3b and Supplementary Fig. S4a). Interestingly, the three modalities with cells from freshly dissociated tissue (Fresh_droplet_PolyA, Fixed_droplet_Probe and Fixed_in-situ_PolyA) consistently favoured immune cell types. In contrast, the two modalities with cells or nuclei from stored tissue blocks (FFPE_droplet_Probe and Nuclei_droplet_PolyA) consistently favoured non-immune cell types, which agrees with the *in situ* spatial transcriptomics sample.

We then investigated the immune and non-immune populations individually (Fig. 3c and Supplementary Figs S4b and c, and S5), to explore cell types at higher granularity. In the immune population, again the three modalities with cells from freshly dissociated tissue (Fresh_droplet_PolyA, Fixed_droplet_Probe and Fixed_in-situ_PolyA) were relatively consistent for a lymphoid derived cell type (NK/T cells), which represented over half the cells in the population. This cell type is less present in the remaining two modalities (FFPE_droplet_Probe and Nuclei_droplet_PolyA), which is again consistent with the *in situ* spatial transcriptomics sample. FFPE_droplet_Probe has a broader representation of cell types, while Nuclei_droplet_PolyA greatly favours a fetal myeloid cell type (Hofbauer cells). In the non-immune cell populations, there is again consistency in the three modalities with cells from freshly dissociated tissue (Fresh_droplet_PolyA, Fixed_droplet_Probe and Fixed_in-situ_PolyA), in which over 90% of cells in this population are STB in each modality. There is, however, variation in the cell types in the remaining 10% of the non-immune population. Of note is the lack of myofibroblasts in these modalities, a cell type well represented in the *in situ* spatial transcriptomics sample. FFPE_droplet_Probe and Nuclei_droplet_PolyA both also have high representation of STBs; however, both have much broader representation of cell types, consistent with the *in situ* spatial transcriptomics sample (Supplementary Fig. S5).

These results showed good consistency between samples and underscore the need to understand the limitations of different methods of tissue processing.

### Protein coding gene expression variance

**Figure 4.**
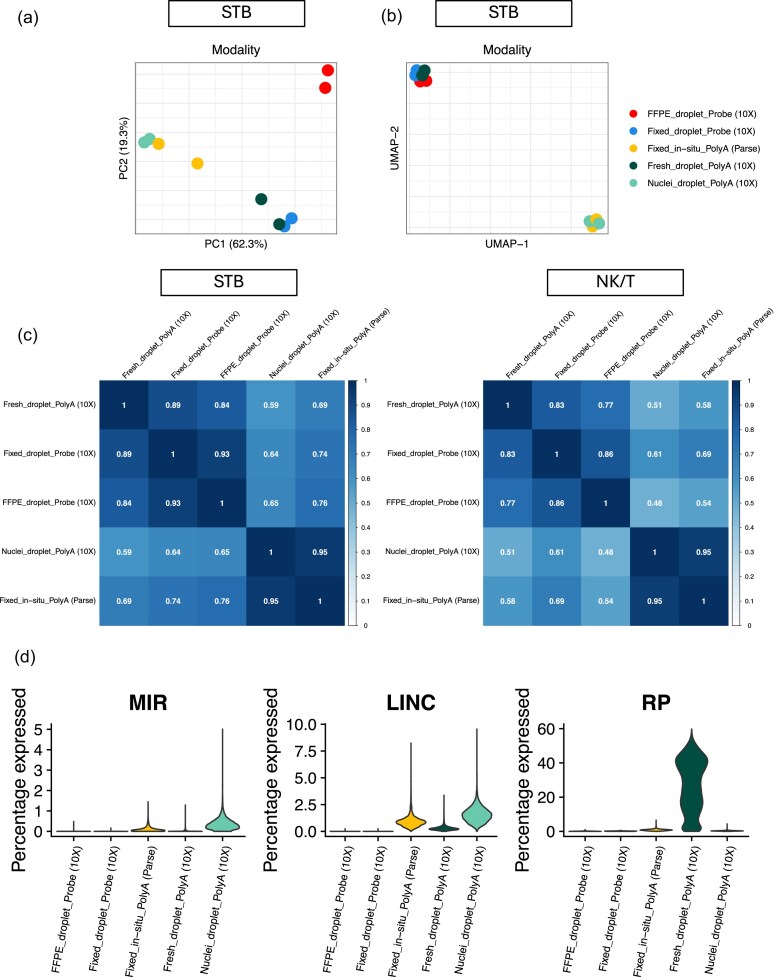
Gene expression differences across modalities. (**a**) Principal Component Analysis of pseudobulked STBs expression of protein coding genes, per library, coloured by modality. (**b**) UMAP of pseudobulked STB expression of protein coding genes, per library, coloured by modality. (**c**) Correlation matrix of per cell-type pseudobulked protein coding gene expression of STB and Natural Killer/T-cell across modalities. (**d**) Distribution of percentage expression in cells of LINC, MIR, and RP genes per modality.

To assess how different modalities varied transcriptionally, we first applied dimensionality reduction techniques. We selected the cell type that is most consistently represented across all libraries (STB). To maintain consistency with most common analytical approaches we independently pseudobulked the expression profiles per library and subsetted the non-probe-based modalities to only include the genes that were present in the probe sets. This ensured we were looking at protein coding genes, avoiding biases caused by differences in gene sets explored. We then performed a principal component analysis, using the top 2000 most variable genes per library (Fig. 4a). The first principal component (PC1) accounted for 62.3% of the variance between libraries with the Nuclei_droplet_PolyA modality showing the largest amount of separation from the other modalities, followed by the FFPE_droplet_Probe and Fixed_in-situ_PolyA (Supplementary Fig. S8). The second principal component (PC2) accounted for 19.3% of the variance with FFPE_droplet_Probe showing the largest amount of separation, perhaps illustrating the transcriptomic effect of the extensive processing steps FFPE samples undergo. We also show small separation between Nuclei_droplet_PolyA and Fixed_in-situ_PolyA, as well as between Fixed_droplet_Probe and Fresh_droplet_PolyA. Intriguingly, despite the low variance between samples in each modality, the two libraries from Fixed_in-situ_PolyA and Fresh_droplet_PolyA show considerably higher separation across the two principal components compared to the separation between libraries produced by any other modality. This is echoed in the larger variance in sensitivity between biological replicates seen in Fixed_in-situ_PolyA and Fresh_droplet_PolyA, which may indicate variation in output consistency between modalities. We then generated a UMAP, using the top 5 principal components (accounting for 95% of variance) (Fig. 4b), in which we observed the Nuclei_droplet_PolyA clustering with the Fixed_in-situ_PolyA with clear separation from a cluster containing the remaining three modalities.

Furthermore, to quantify the similarity of cell-type-specific gene expression profiles among modalities, we selected the two cell types that are most consistently represented across modalities (STB and NK/T cells). We independently pseudobulked their expression profiles per modality for each cell type. We again subsetted the non-probe-based modalities to only include the genes that were present in the probe sets, and therefore protein coding. We then generated a correlation matrix for each cell type from the normalized counts across modalities (Fig. 4c). This allows us to assess the similarity of gene expression profiles from the same cell types across modalities using the same gene set. As expected, there was high correlation (Pearson coefficients: 0.77–0.93) between the three whole cell droplet-based modalities (Fresh_droplet_PolyA, Fixed_droplet_Probe and FFPE_droplet_Probe). The highest correlation (Pearson coefficient: 0.95 for both STB and NK/T), however, was between Fixed_in-situ_PolyA and Nuclei_droplet_PolyA. This echoes the PCA and UMAP results detailed above and indicates potential bias towards nuclear transcripts for the Fixed_in-situ_PolyA.

### Alternative gene expression features by modality

We also sought to understand the variance in the alternative genes detected between the different modalities. We queried the percentage of counts in cells per modality for three different classes of genes, which are not traditionally investigated in most transcriptomic analyses: RP, MIR, and LINC (Fig. 4d). As MIR and LINC genes are non-coding genes and RP genes are highly conserved with low variance in expression, they are frequently excluded from analysis. By quantifying their prevalence in our results, we can assess the proportions of transcripts that are likely to be commonly excluded from investigations. Alternatively, where LINC and MIR genes are of particular interest, our quantification provides information on which modalities provide best coverage of these gene types. Fresh_droplet_PolyA had very high proportion of RP genes compared to the rest of the modalities with a significant number of cells reaching and even exceeding 40% of counts. The highest percentage of MIR and LINC genes was observed in Nuclei_droplet_PolyA, which is expected as both these gene classes are of nuclear RNAs. However, significant levels were also detected in Fixed_in-situ_PolyA. Unsurprisingly, both probe-based approaches (Fixed_droplet_Probe and FFPE_droplet_Probe) had almost no counts for any of the three gene classes, as probes targeting these genes are largely absent from their probe sets.

### Differential gene expression across modalities

**Figure 5.**
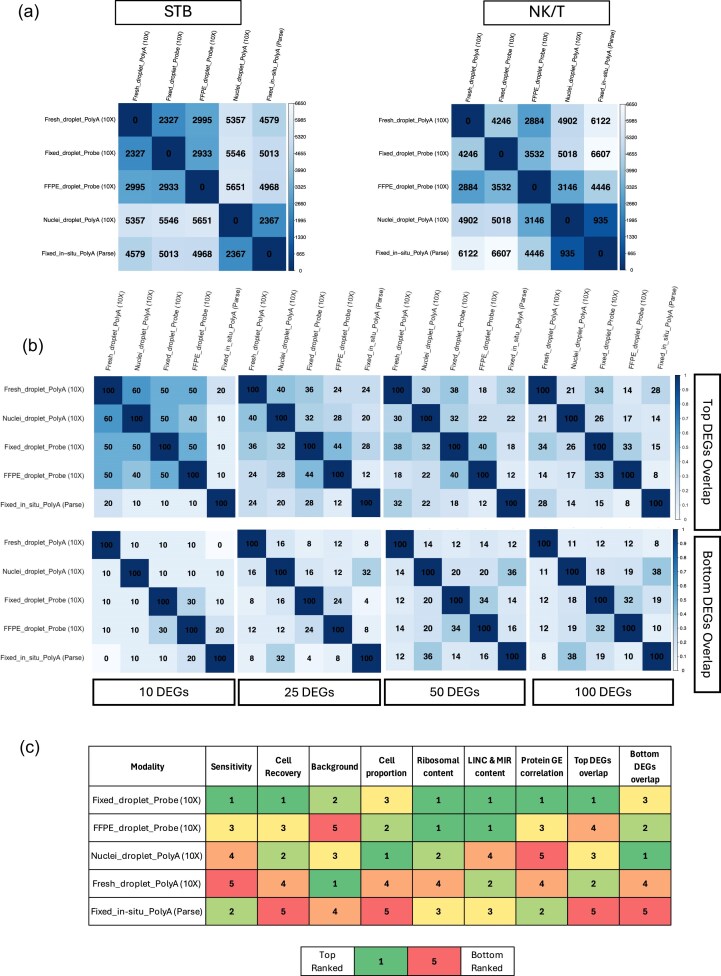
Differential gene expression analysis variance. (**a**) Matrices illustrating number of DEGs from pairwise comparison between modalities for STB and NK/T cells. Supplementary Fig. S9 presents matrices of equivalent pseudobulk DEG analysis. (**b**) Matrixes illustrating proportional overlap of 10, 25, 50, and 100 most over and under expressed genes by average log_2_-fold change (adjusted *P*-value <.05), when comparing STBs between biological replicates, within each modality. (**c**) Ranking of each modality by its performance on sensitivity, cell recovery, background amount, cell proportion (as judged by similarity to *in situ* spatial transcriptomics), RP content, LINC, and MIR gene content, protein coding gene expression (GE) correlation and overlap of top and bottom DEGs. The ranking approach is explained in the ‘Comparative ranking of modalities’ section.

We globally quantified the technical variance between modalities comparing the number of DEGs across them. To do this, we again subsetted the STBs and NK/Ts and performed pairwise DEG analysis between modalities, for each of the two cell types independently (Fig. 5a). For both cell types, we report relatively few DEGs between Fixed_in-situ_PolyA and Nuclei_droplet_PolyA, which echoes our previous results (Fig. 2). Intriguingly, where for STBs, Nuclei_droplet_PolyA has the most DEGs compared to the other whole-cell, droplet based approaches, with Fixed_in-situ_PolyA having second most, for NK/Ts, this trend flips, with Fixed_in-situ_PolyA has more DEGs compared to the three whole-cell, droplet based approaches with Nuclei_droplet_PolyA having second most. This likely suggests a cell-type-specific component to the technical variance between modalities.

### Commonalities of differential gene expression analysis

To explore how different single cell modalities impact the results of a typical differential gene expression analysis, we compared the same cell type (STBs) between two donors (biological replicates) in each modality for the top upregulated and downregulated genes. We filtered the resulting DEGs, retaining only those with an adjusted *P*-value below .05 and ranked them by descending log_2_-fold change (Supplementary Fig. S10). We subsequently explored the percentage of overlapping DEGs when looking at the top (most upregulated) and bottom (most downregulated) 10, 25, 50, and 100 DEGs across modalities (Fig. 5b). This analysis thus identifies biological variation among replicates that is recovered across modalities.

We show that when exploring the top 10, Fixed_in-situ_PolyA has considerably lower overlapping DEGs across modalities. Athough this pattern does persist, as we look at increasing numbers of DEGs, the overlap does increase. Additionally, there is consistently high overlap between the two probe-based approaches (FFPE_droplet_Probe and Fixed_in-situ_PolyA) in both top and bottom DEGs. For the top 50 and 100 DEGs, there is notable overlap between Fixed_droplet_Probe and Fresh_droplet_PolyA, supporting our findings in the PCA (Fig. 4a). At the top 10 and 25 DEGs, we also observe notable overlap between Fresh_droplet_PolyA and Nuclei_droplet_PolyA. Finally, when exploring the bottom 25, 50, and 100 DEGs there is consistently higher overlap between Nuclei_droplet_PolyA and Fixed_in-situ_PolyA. It is important to note that as previously presented (Figs 2b and c, and 5a) the overlap of ranked DEGs in other cell types, present in too few numbers for us to interrogate, could somewhat differ.

### Overview of different modalities

In order to provide a summary of key metrics that are of significant importance for experimental design and analytical approaches, we present an overview of the comparable performance of the modalities explored across nine variables (Fig. 5c), with our ranking approach described in the ‘Comparative ranking of modalities’ section. This can be used as a simple guide to the design of future experiments according to sample, storage, and processing characteristics.

## Discussion

We present a quantification and exploration of the technical variance between five single cell and nuclei transcriptomic modalities on a challenging tissue, the placenta. Due to this challenging nature of the placenta and the technical variance in cell-type sampling, we have focused our analyses on cell types that are present in sufficient numbers across all five modalities.

Although all modalities yielded reasonable results for both biological replicates, the highest consistent sensitivity is achieved with Fixed_droplet_Probe. Due to the probe-based transcript capture excluding non-protein coding genes and other gene types, the supplier’s recommended sequencing depth is also half of that of the polyA modalities, making it a more cost-effective solution. An important consideration, however, is the absence of some genes from these probe sets. Probes that target genes with hypervariable regions such as human leukocyte antigen (HLA*), T-Cell receptor variable (TRV*), and immunoglobulin variable (IG*) genes are absent. For some biological questions this can be a serious limitation. For example the expression of HLA-G, is a marker gene for extravillous trophoblasts (EVTs), is not interrogated by these probe sets. Custom probes for specific absent genes of interest can be used, but as with any custom addition, may yield suboptimal results. For exploratory investigations and for questions with immunological nuance, this poses a challenge.

A key motivation for utilising single cell transcriptomics is to quantify cell-type proportions and resolve heterogenous gene expression among cell types. Thus, it is important to evaluate the capacity and potential bias of each modality to transcriptionally represent the different cell types. Our results delineate differences among modalities in cell-type proportions. Notably, we identify tissue processing as the main driver of differing cell-type proportions across modalities. The three modalities with cells from freshly dissociated tissue have broadly comparable cell-type proportions, both at the lower and higher levels of granularity of cell-type annotation. These are distinct from the two modalities with cells and nuclei from stored tissue blocks, either fresh frozen or FFPE, as well as the *in situ* spatial transcriptomics sample with relatively well-maintained *in vivo* cell-type proportionality. This is likely due to the requirement for cells from freshly dissociated tissues to be highly viable (optimally over 90%) and free from red cell contamination. As such, following an enzymatic and mechanical dissociation, these cell suspensions are subjected to red cell lysis and magnetic-activated cell sorting (MACS) for dead cell removal. These processing steps undoubtedly affect cell-type proportions, with more sensitive cell types adversely affected by red cell lysis and more resilient ones favourably enriched by the dead cell removal. Previous studies have reported different cell-type proportions from single cell analyses from freshly dissociated placentas, using alternative dissociation protocols and samples with different biological variables. This further supports our claim that sample processing should be central to considerations of experimental design [15].

The two modalities with cells and nuclei from stored tissue blocks do not require any cell enrichment steps, atlthough it is possible that either formalin fixation or snap freezing affect cell-type proportions differently, as enzymatic and mechanical dissociations processes may also do. Hence, all modalities may have biases that need to be understood. To ameliorate for these biases and to ensure maximal proportional representation, Admati *et al*. [29] and Garcia-Flores *et al*. [30] used both cells derived from freshly dissociated samples as well as nuclei from snap frozen samples from the same placentas. It is a valuable strategy, but we note that in other tissues more inherently free from red cell contamination and with highly viable cells directly post dissociation, biases may be less pronounced. Ultimately, the variation in cell-type proportions can have a severe impact on studies investigating particular cell types, as certain modalities may completely exclude cell types well represented *in vivo*, such as the absence of CTBs from the plate-based Fixed_in-situ_PolyA, or placental myofibroblasts in the modalities requiring freshly dissociated tissue likely due to the shape of this cell type.

Understanding the levels of variation in the protein coding transcriptomes of the same cell types across modalities allows us to identify which ones produce equivalent results and which, if any, do not. As expected, the Nuclei_droplet_PolyA had the lowest level of correlation with the other droplet-based modalities, likely due to the lack of cytoplasmic mRNAs inherent in this modality. Surprisingly however, the Fixed_in-situ_PolyA and Nuclei_droplet_PolyA modalities showed the highest level of correlation. This is best observed in the PCA and UMAP presented.

This may suggest that although the Fixed_in-situ_PolyA modality as currently offered by Parse Biosciences and other providers is meant to capture both cytoplasmic and nuclear mRNA, there may be a bias in their chemistry favouring nuclear transcripts. This suggestion is somewhat supported by the observation that the Fixed_in-situ_PolyA had considerably higher levels of expression of non-protein coding nuclear genes (MIR, LINC) than the three whole-cell droplet-based modalities. One potential explanation, which we have not yet explored, is that the specialized fixation and permeabilization in Fixed_in-situ_PolyA, required for cells to act as a reaction chamber through multiple rounds of transcriptomic indexing, also permits cytoplasmic mRNA to escape the cellular boundary. This again would suggest an effect intrinsic to this technology, which may not be resolved either by new versions of chemistry or alternative commercial providers, (e.g. Scale Biosciences). This is an important consideration for studies where cytoplasmic transcripts may be of particular interest, as recently demonstrated in placentas complicated by preeclampsia [15].

Beyond the potential for the resolution of biological samples, single cell transcriptomics is now commonly used in case-control comparison studies to detect disease signatures within and across cell types [2]. We found that modality will impact the proportions of cell types and their comparison. This could render changes in proportions that are not biological but method-dependant and should be taken into consideration in case-control studies. Moreover, the reliability of each method to detect disease markers is of paramount importance. In as much as reliability can be assessed by the reproducibility of DEGs detected across modalities, Fixed_in-situ_PolyA is notably less able to detect DEGs found by other modalities. This effect is increasingly pronounced when exploring the highest and lowest ranked DEGs by fold change. This suggests that even when DEGs are commonly detected across single cell modalities, their ranked position by fold change varies significantly, potentially shifting markers of disease in case-control comparisons. Notably, there is also a pattern of high concordance between Fixed_in-situ_PolyA and Nuclei_droplet_PolyA in the most downregulated genes.

An additional element that should be considered for the experimental design of studies employing single cell transcriptomics is the protocol complexity and its downstream analysis. The droplet-based modalities in this study, offered by 10X Genomics, require specialized, expensive instrumentation that may not be available to many researchers. However, this instrumentation is frequently available in genomic core facilities. Conversely, the plate-based *in situ* combinatorial indexing approach presented, offered by Parse Biosciences, requires simpler instrumentation, frequently already present in laboratories such as thermocyclers and multi-channel pipettes. However, this modality requires considerably more ‘hands-on’ time with multiple pipetting steps, introducing higher chances of processing errors. Furthermore, as this modality requires the pooling of samples as part of the cellular indexing, the resulting libraries are a composite of all samples processed in a batch. As such, it is impossible to sequence one sample at a time, or simply re-sequencing a single sample on its own, should there be an imbalance of molarity per sample in the resulting composite library. In contrast, droplet-based approaches mainly generate one sequencing library per sample.

Taken together, we present a comprehensive analysis of the technical variance between different single cell transcriptomic modalities in a challenging tissue, the human placenta, highlighting the strengths and weaknesses inherent in each modality (Fig. 5c). We provide novel and essential considerations required for experimental design and analytical approaches in single cell transcriptomics, some relevant to the identification of disease markers. These findings are likely applicable to other challenging tissues, whether due to inherent biological characteristics, sampling approaches available or degrading effects of pathology.

## Supplementary Material

lqag079_Supplemental_File

## Data Availability

The data underlying this article are available in the NCBI Gene Expression Omnibus repository (https://www.ncbi.nlm.nih.gov/geo/) with accession numbers GSE330886 and GSE310326. Processed Seurat object and code used for the analysis are available on Zenodo (Seurat object: https://doi.org/10.5281/zenodo.20543857, Code: https://doi.org10.5281/zenodo.20700773).
