# Supplementary material for: The challenges of single cell transcriptomics on difficult human tissue: the placenta
Source: NAR Genom Bioinform. 2026 Jul 16;8(3):lqag079. doi: 10.1093/nargab/lqag079 (PMC13373321; doi:10.1093/nargab/lqag079)
Supplement: lqag079_Supplemental_File [file lqag079_supplemental_file.pdf]

Extended Data

The challenges of single cell transcriptomics on difficult human tissue: the placenta

AUTHORS

Theodoros Xenakis[1*], George T Hall[1], Jose J Moreno-Villena[1], Sara L Hillman[3,4], Yara E Sanchez-Corrales[1] and Sergi Castellano[1,2] *

* To whom correspondence should be addressed. Theodoros Xenakis and Prof Sergi Castellano. Email: t.xenakis@ucl.ac.uk and s.castellano@ucl.ac.uk

This PDF file includes:

Extended Figure 1. Sensitivity variance between donors.

Extended Figure 2. Performance metrics per biological replicate.

Extended Figure 3. Marker gene expression of annotated cells.

Extended Figure 4. Cell type proportions by biological replicate.

Extended Figure 5. Cell type proportion differences**.**

Extended Figure 6. Variance between biological replicates and across modalities.

Extended Figure 7. Uniform Manifold Approximation and Projections (UMAP) of total integrated dataset.

Extended Figure 8. Average Euclidian distances between libraries on PC1 of Principal Component Analysis of gene expression profiles of syncitiotrophoblast cells (STBs).

Extended Figure 9. Differential gene expression analysis across modalities.

Extended Figure 10. Ranking of differentially expressed genes per modality.

Extended Table 1. Gene expression markers used for cell-type annotation in single cell transcriptomic libraries and *in situ* Spatial Transcriptomic sample.

Extended Table 2. Sequencing metrics.

Extended Table 3. Cell numbers per cell type and modality.

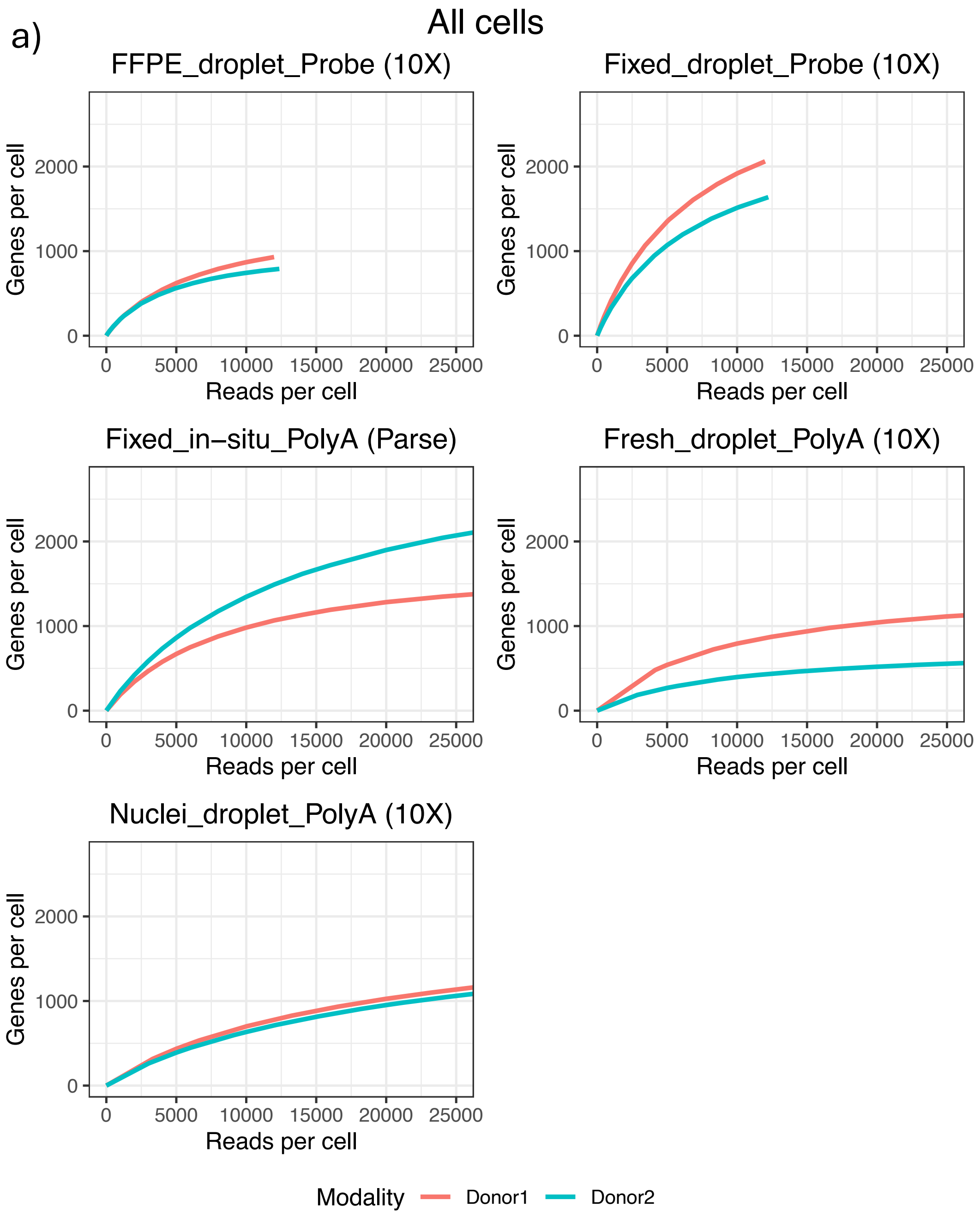

Extended Figure 1. Sensitivity variance between donors. **a)** Sensitivity, as illustrated per donor, for each modality using the relationship between median genes per cell and median reads per cell. (sequencing depth shown for each modality is to the manufacturers minimum recommendation + 5%).

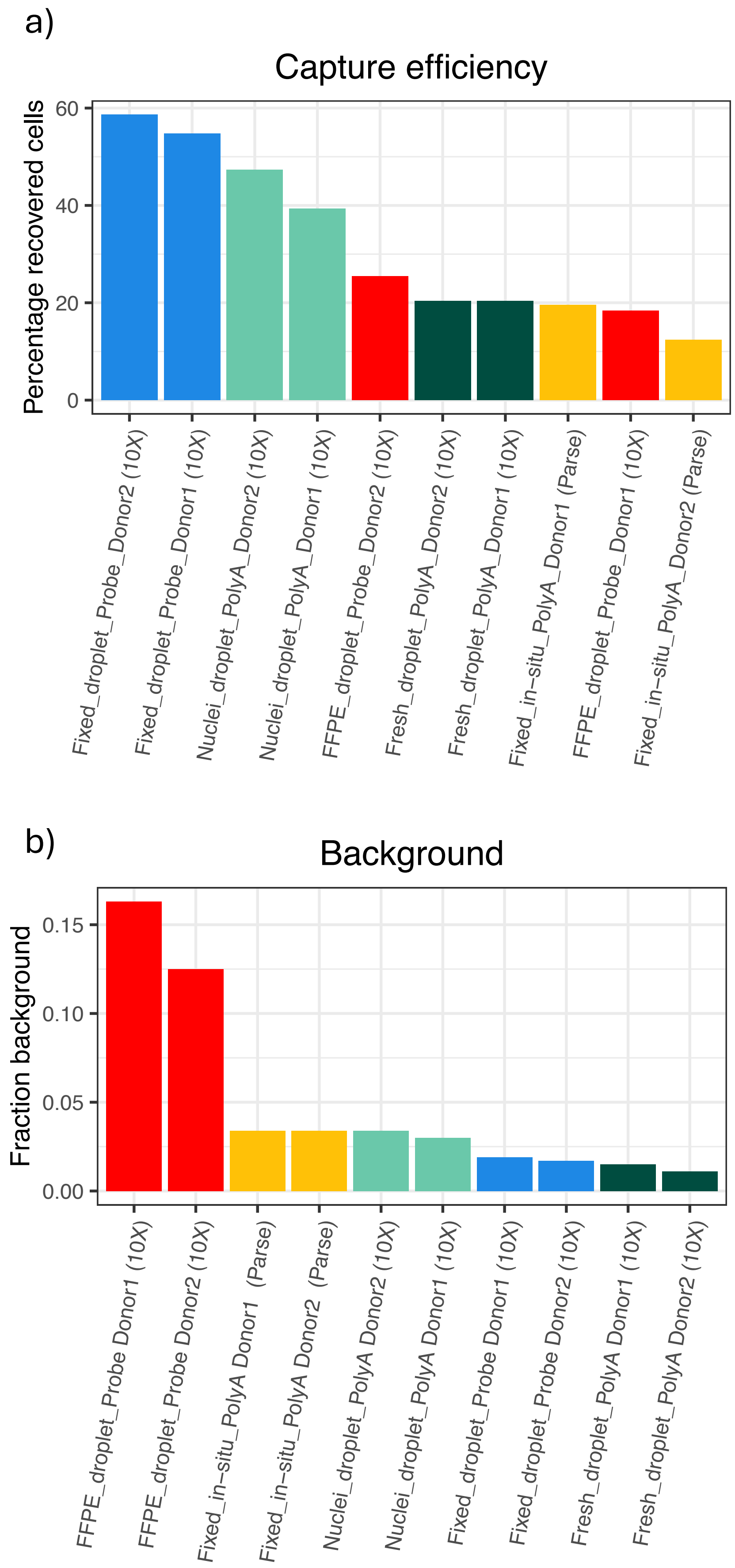

Extended Figure 2. Performance metrics per biological replicate. **a**) Capture efficiency, showing the percentage of cells used for analysis post QC filtering in relation to cells inputted for each biological replicate. **b**) Background, illustrating per biological replicate the fraction of reads removed by the computational tool used for removing reads assigned to ambient RNA.

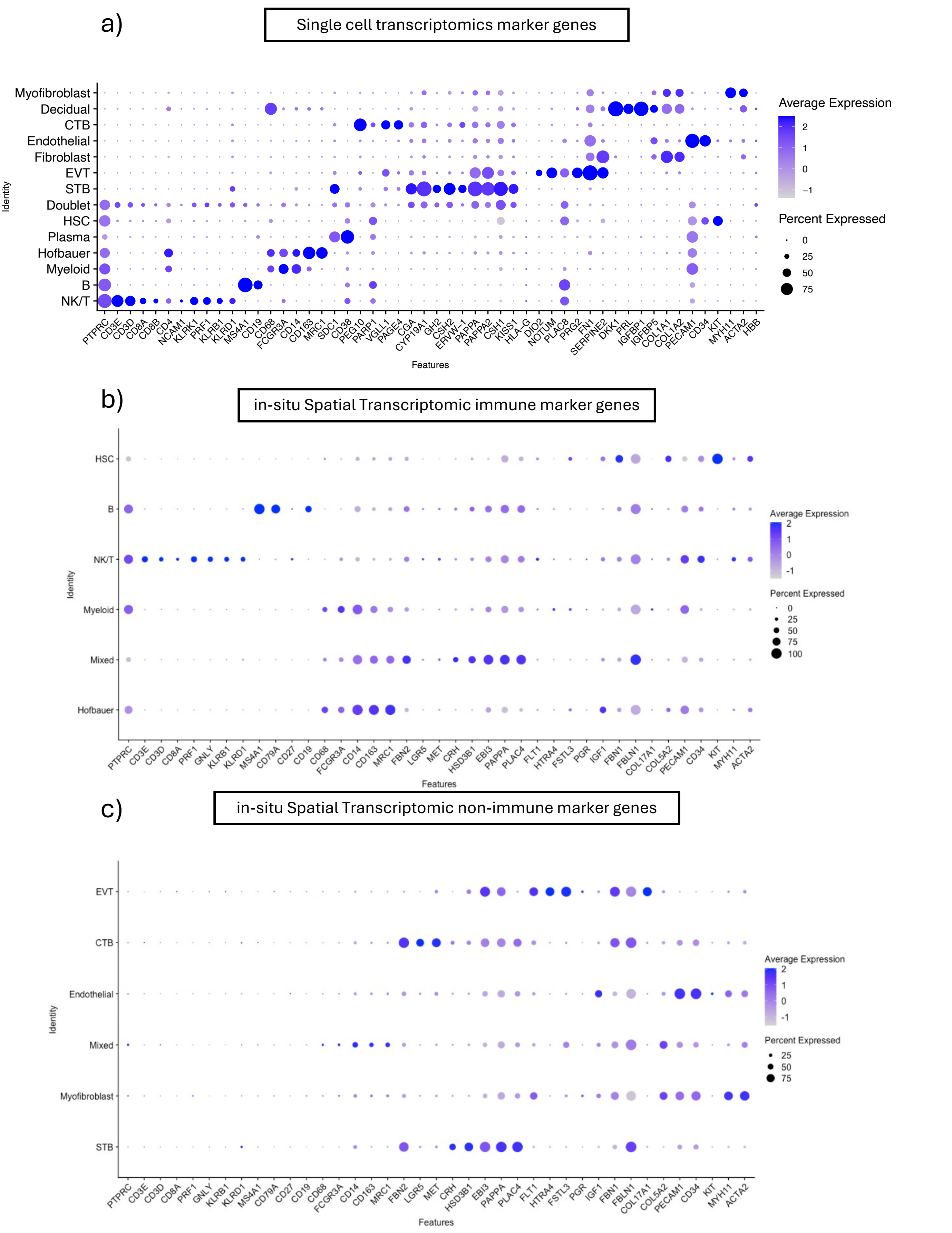

Extended Figure 3. Marker gene expression of annotated cells. **a**) Dotplot illustrating marker gene expression of annotated cell types in combined dataset including all five modalities. **b**) Dotplot illustrating marker gene expression of annotated cell types in immune compartment of in-situ Spatial Transcriptomic sample. **c**) Dotplot illustrating marker gene expression of annotated cell types in non-immune compartment of in-situ Spatial Transcriptomic sample.

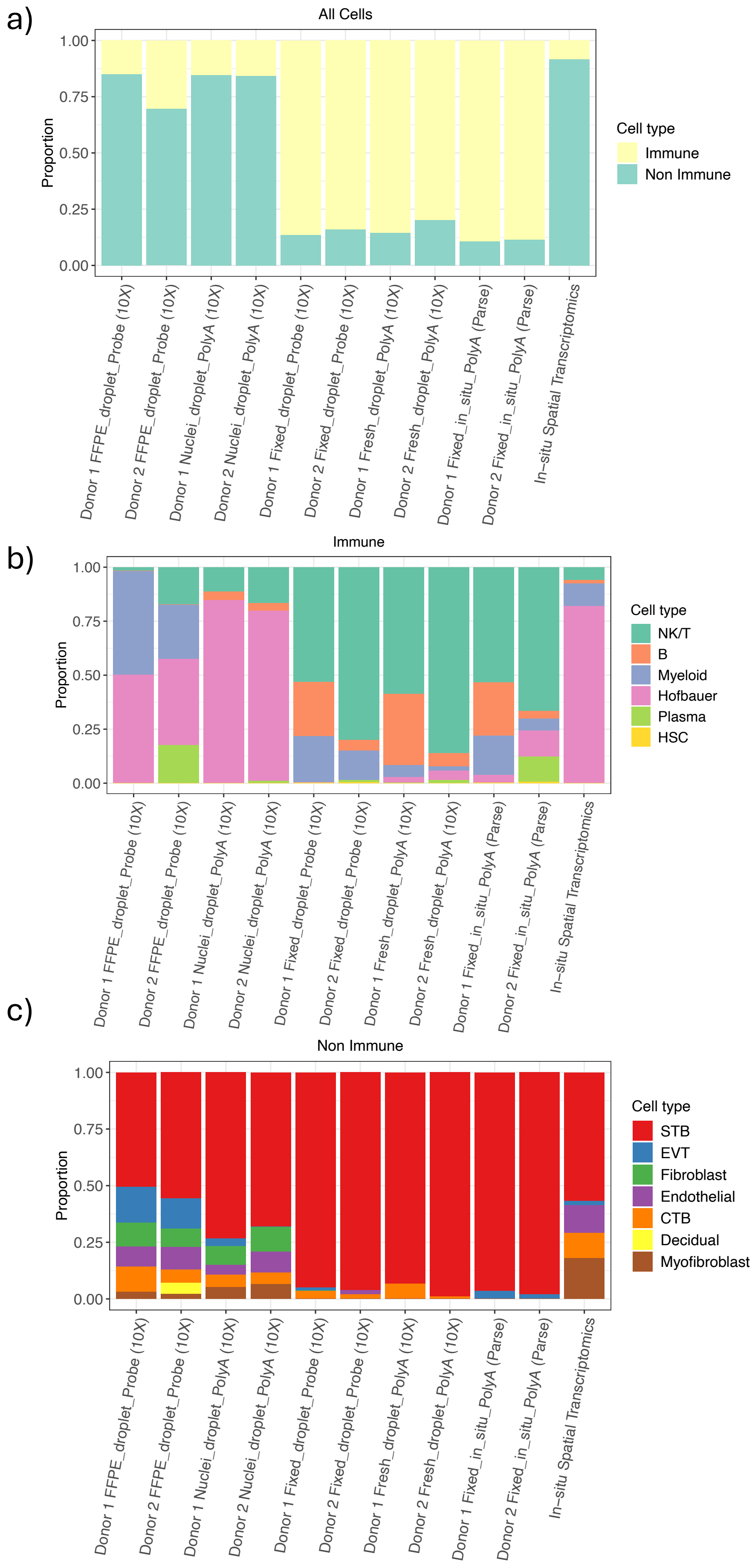

Extended Figure 4. Cell type proportions by biological replicate. **a**) Relative proportion of cells annotated as immune and non-immune cells by biological replicate and modality. Relative proportions of cell annotated within the **b**) immune and **c**) non-immune cell types by biological replicate and modality.

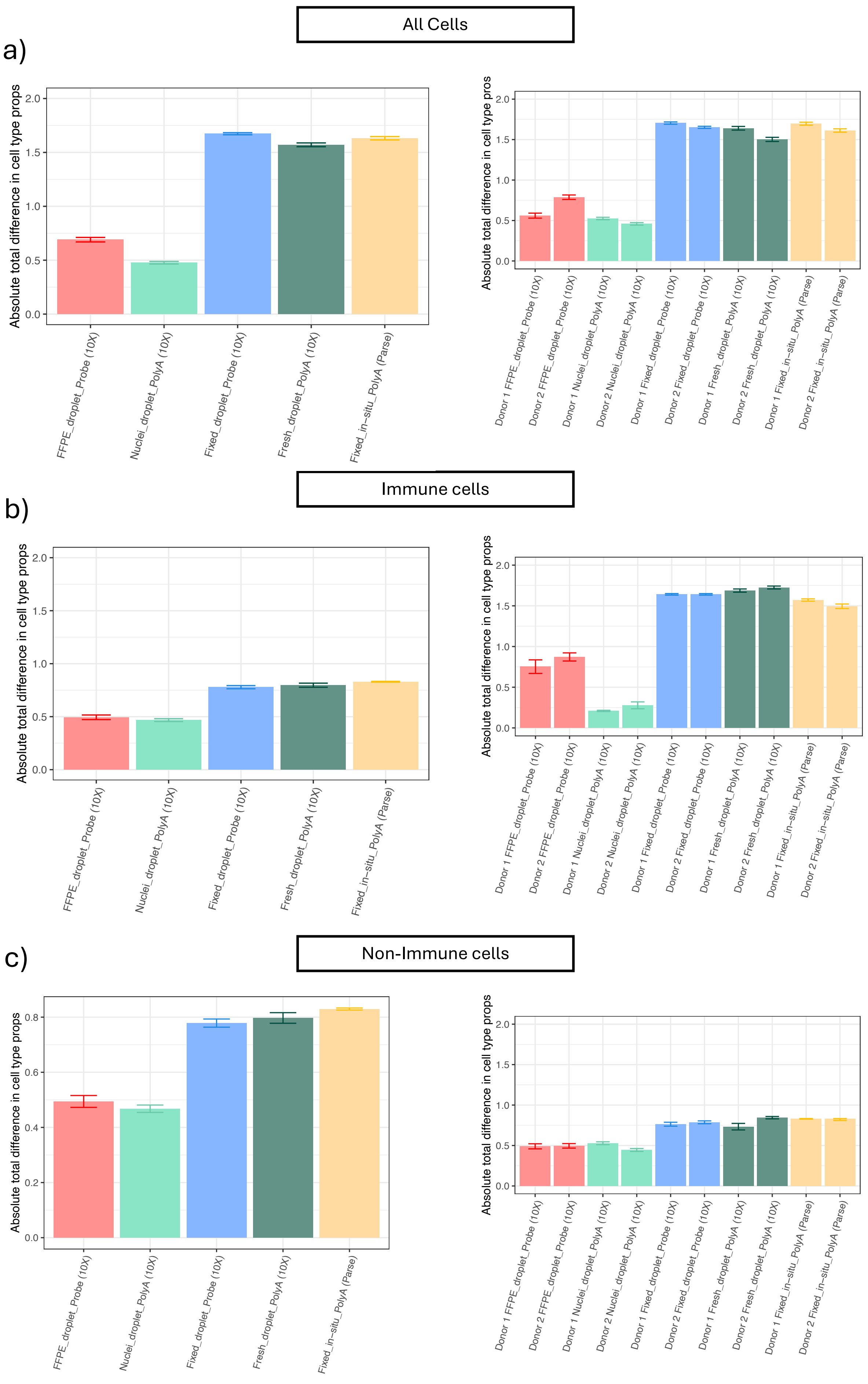

Extended figure 5. Cell type proportion differences**.** The sum of absolute differences between each modality's cell type proportions and those of the *in situ* spatial transcriptomics sample, per modality and library, for **a)** All cell types **b)** Immune cel types and **c)** Non-Immune cell types. Whiskers indicated range of 95% confidence interval.

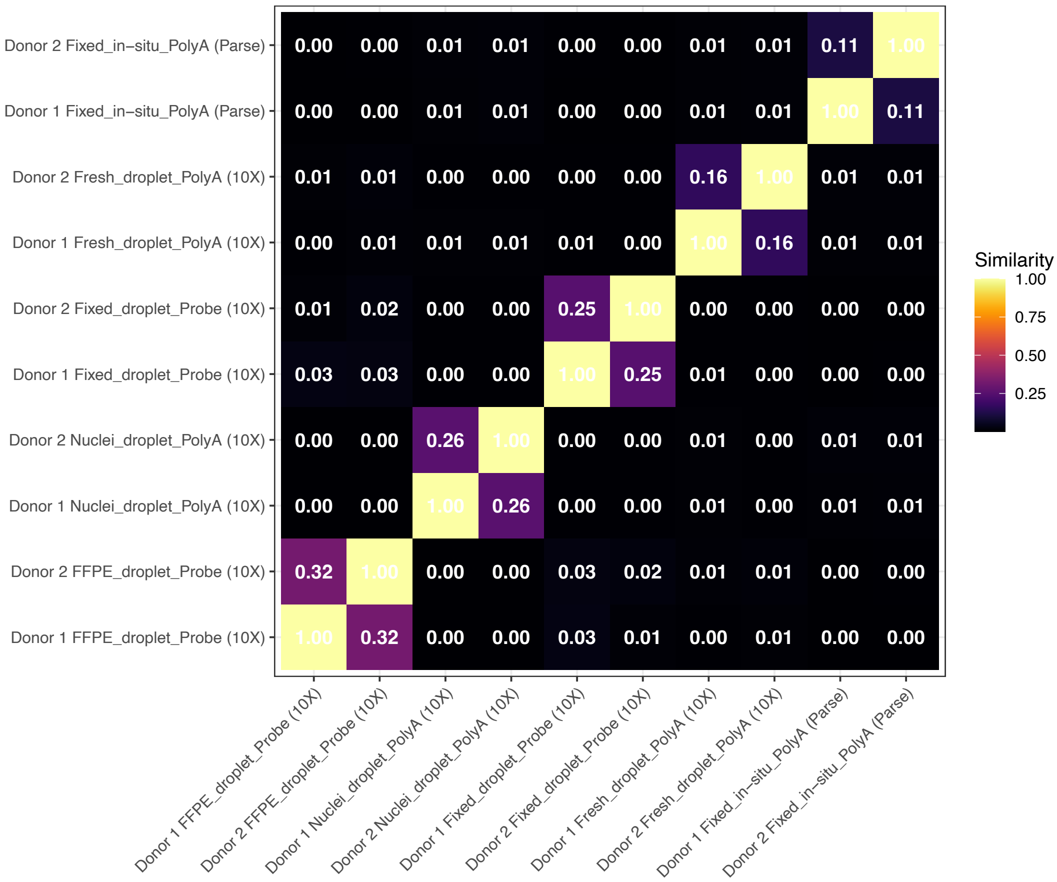

Extended Figure 6. Variance between biological replicates and across modalities. Proportion of cells/nuclei in regions not dominated by a single library, from pairwise PCA-based differential abundance using Dawnn between all pairs of libraries.

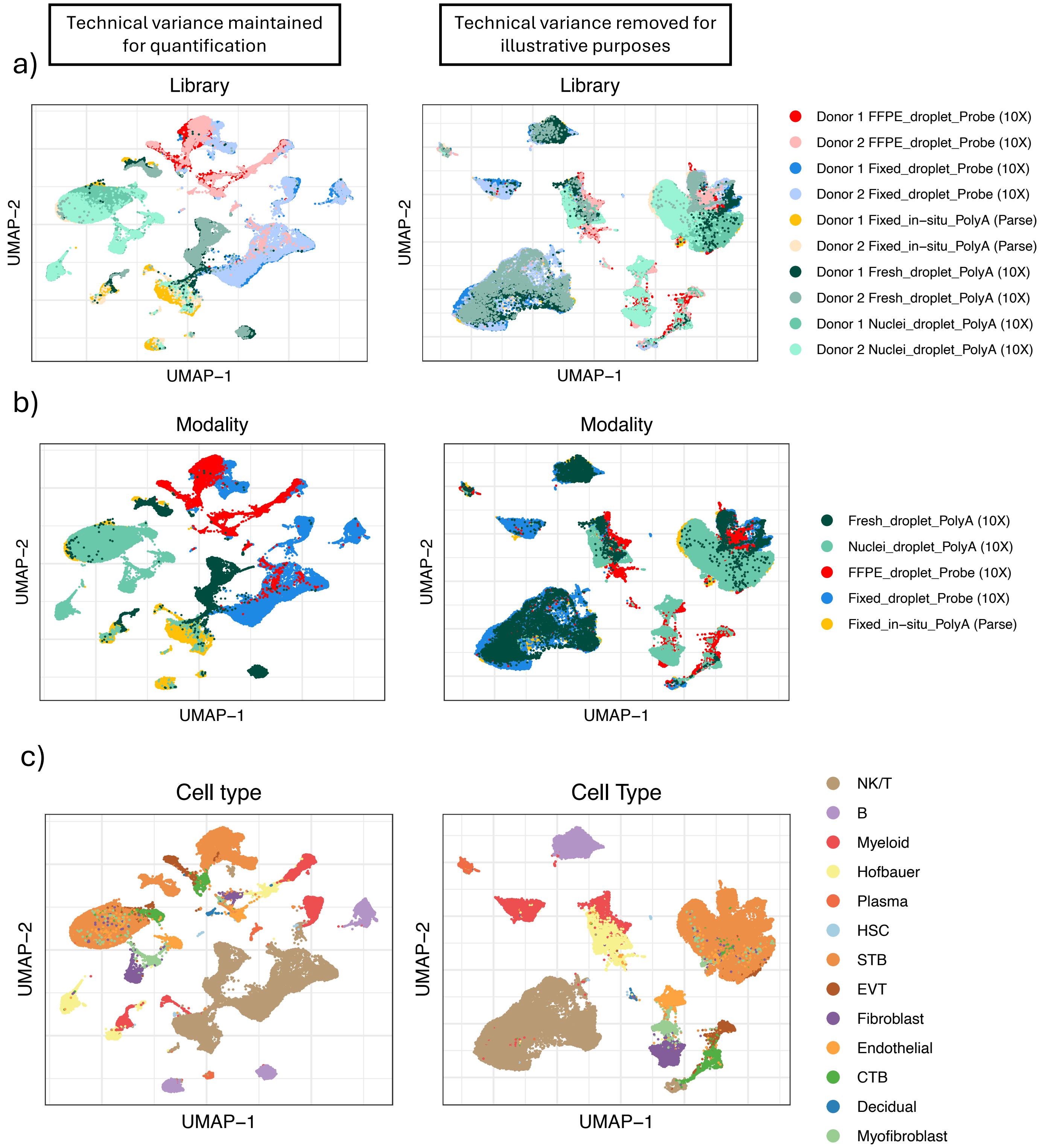

Extended Figure 7. Uniform Manifold Approximation and Projections (UMAP) of total integrated dataset. On the left, with technical variance maintained for interrogation in this manuscript and, on the right, with technical variance removed, masking the potential advantages and disadvantages of each modality. Coloured by **a)** library **b)** modality and **c)** cell type.

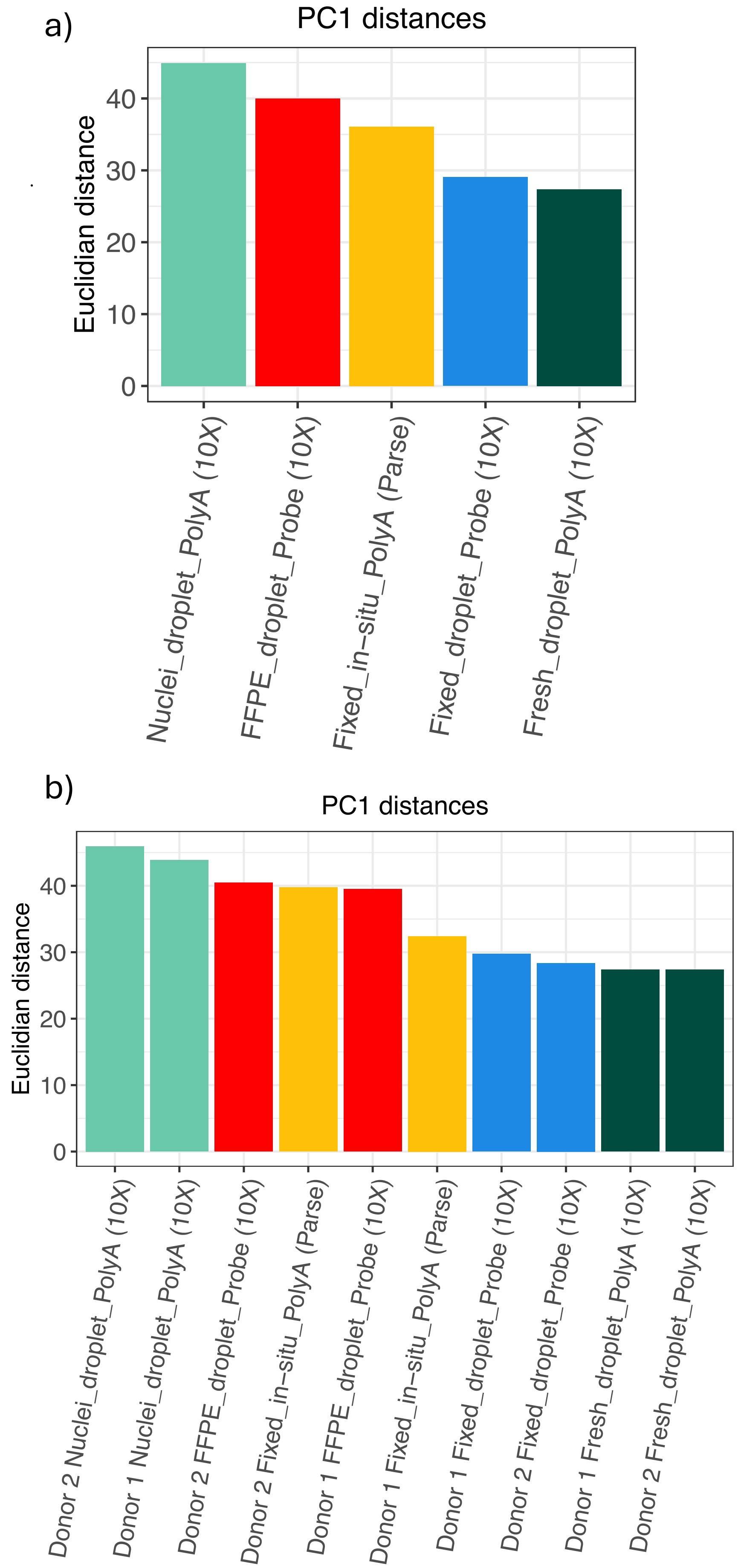

Extended Figure 8. Average Euclidian distances between libraries on PC1 of Principal Component Analysis of gene expression profiles of syncitiotrophoblast cells (STBs). **a**) Distances averaged by modality. **b)** Averaged per library.

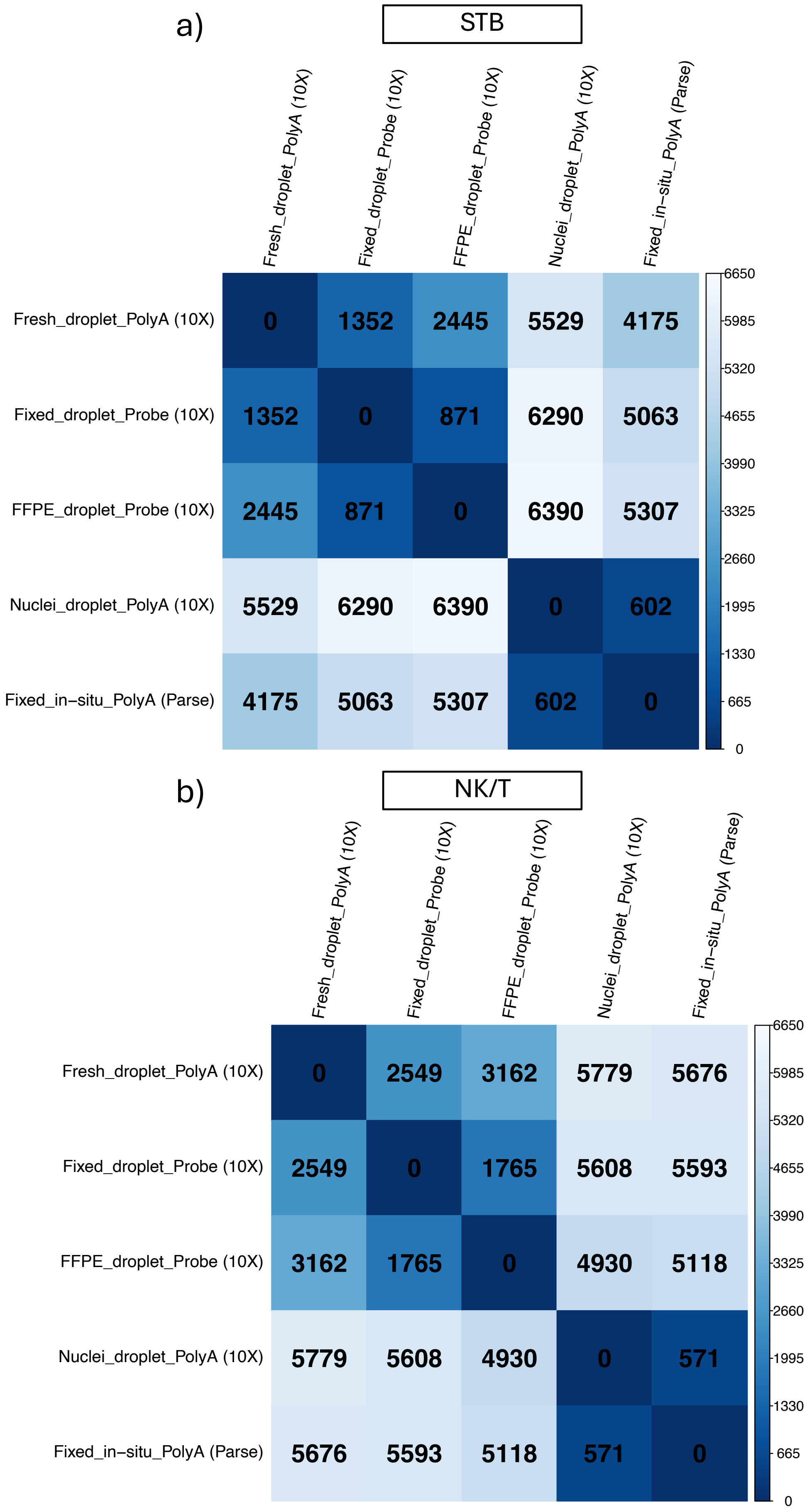

Extended Figure 9. Differential gene expression analysis across modalities. Matrices illustrating number of DEGs from pairwise comparison using pseudobulking and DESeq2 between modalities for **a)** STB and **b)** NK/T cells.

a)

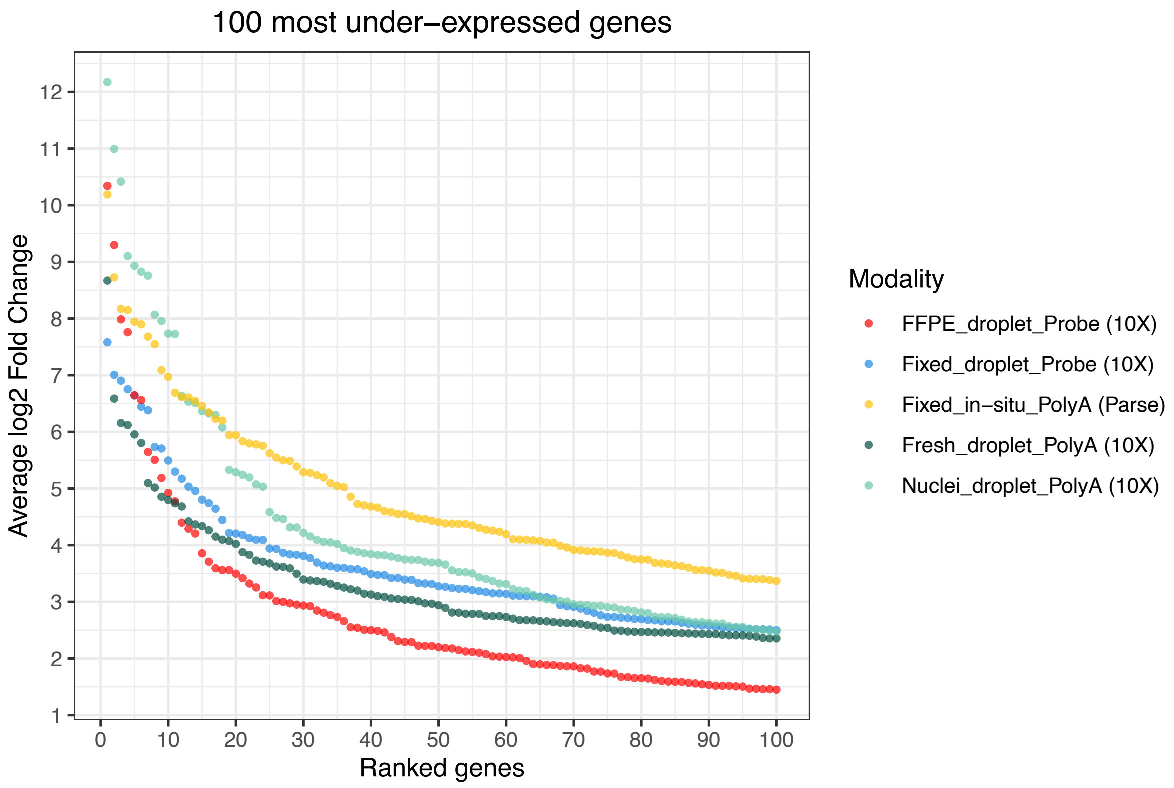

b)

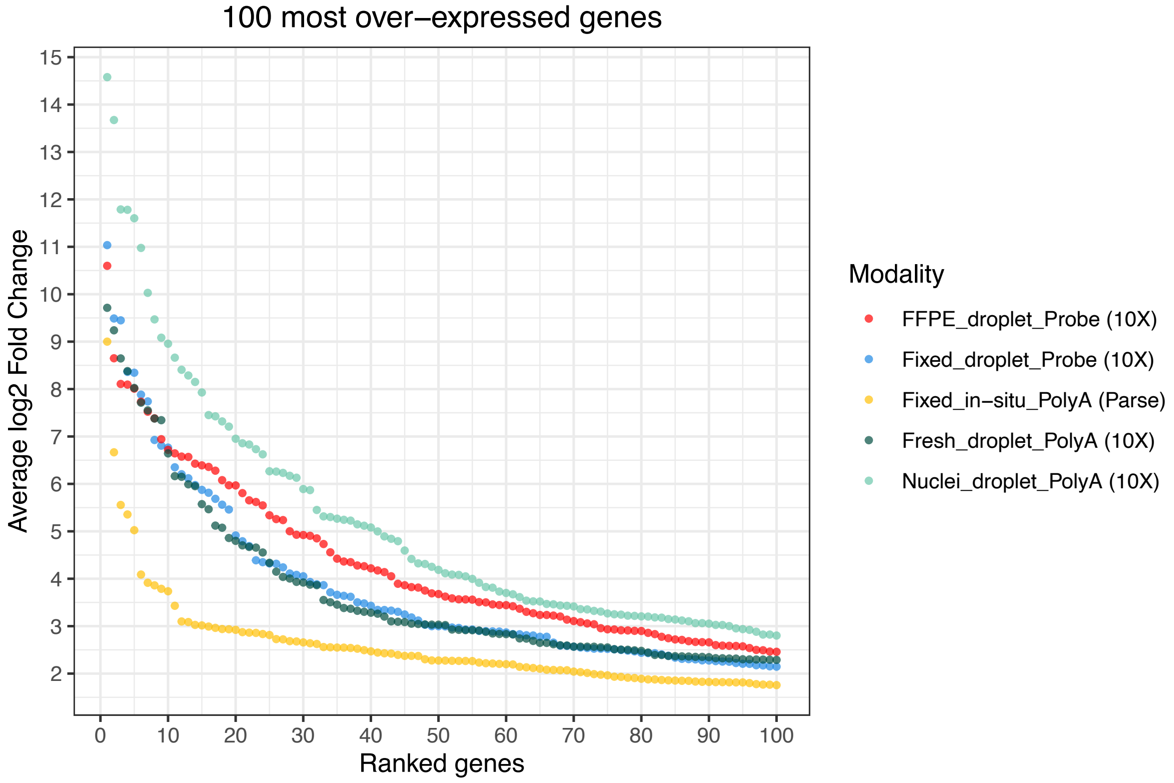

Extended Figure 10. Ranking of differentially expressed genes per modality. **a**) Top 100 over expressed genes ranked by average log2-fold change (adjusted p-value <0.05), when comparing Syncitiotrophoblasts (STBs) between biological replicates, within each modality. **b**) Top 100 under expressed genes ranked by average -log2 fold change (adjusted p-value <0.05), when comparing Syncitiotrophoblasts (STBs) between biological replicates, within each modality.

Single Cell Sequencing Cell Type Markers

NK/T cells	B cells	Myeloid cells	Hofbauer cells	Plasma cells	Haematopoetic stem cells (HSC)	Cytotrophoblast cells (CTB)
PTPRC	PTPRC	PTPRC	PTPRC	PTPRC	PTPRC	PEG10
CD3E	MS4A1	CD68	CD68	SDC1	CD34	PARP1
CD3D	CD19	FCGR3A	FCGR3A	CD38	KIT	VGLL1
CD8A		CD14	CD14			PAGE4
CD8B			CD163			
CD4			MRC1			
NCAM1						
KLRK1						
PRF1						
KLRB1						
KLRD1						

Syncitiotrophoblast cells (STB)	Extravillous trophoblast cells (EVT)	Decidual cells	Fibroblast cells	Endothelial cells	Myofibroblast cells
CGA	PAPPA	DKK1	FN1	PECAM1	COL1A1
CYP19A1	PAPPA2	PRL	SERPINE2	CD34	COL1A2
GH2	HLA-G	IGFBP1	COL1A1		MYH11
CSH2	DIO2	IGFBP5	COL1A2		ACTA2
ERVW-1	NOTUM				
PAPPA	PLAC8				
PAPPA2	PRG2				
CSH1	FN1				
KISS1	SERPINE2				

in situ Spatial Transcriptomic Cell Type Markers

NK/T cells	B cells	Myeloid cells	Hofbauer cells	Plasma cells	Haematopoetic stem cells (HSC)	Cytotrophoblast cells (CTB)
PTPRC	MS4A1	CD68	CD68		PTPRC	FBN2
CD3E	CD79A	FCGR3A	FCGR3A		CD34	LGR5
CD3D	CD19	CD14	CD14		KIT	MET
CD8A			CD163			
PRF1			MRC1			
GNLY						
KLRB1						
KLRD1						

Syncitiotrophoblast cells (STB)	Extravillous trophoblast cells (EVT)	Decidual cells	Fibroblast cells	Endothelial cells	Myofibroblast cells
CRH	PAPPA	PGR	FBLN1	PECAM1	FBLN1
HSD3B1	PLAC4	IGF1	COL17A1	CD34	COL17A1
EBI3	FLT1		COL5A2		COL5A2
PAPPA	HTRA4				MYH11
PLAC4	FSTL3				ACTA2
	FBN1				

Extended Table 1. Gene expression markers used for cell-type annotation in single cell transcriptomic libraries and *in situ* Spatial Transcriptomic sample.

Library	Total sequencing reads	Mean reads/ cell	Cell Barcodes	Cells post-Cellbender	Doublet	Low Quality Cells	Cells post processing	Sequencing saturation
Donor 1 FFPE_droplet_Probe	220,722,175	12,365	17,851	7759	893	3,193	3673	63.70%
Donor 1 Fixed_droplet_Probe	344,727,781	20,390	16,907	15442	286	6,407	8749	60.71%
Donor 1 Fixed_in_situ_PolyA	594,689,927	92,920	6,400	9345	276	3,667	5402	71.50%
Donor 1 Fresh_droplet_PolyA	274,337,560	28,702	9,558	6991	332	2,546	4113	62.74%
Donor 1 Nuclei_droplet_PolyA	243,322,449	30,313	8,027	10,273	637	1,567	8069	38.53%
Donor 2 FFPE_droplet_Probe	253,587,927	13,322	19,035	10003	701	4,180	5122	56.27%
Donor 2 Fixed_droplet_Probe	330,213,709	17,144	19,261	17045	348	4,505	12192	53.66%
Donor 2 Fixed_in_situ_PolyA	202,241,389	38,714	5,224	5698	381	1,740	3577	71.30%
Donor 2 Fresh_droplet_PolyA	311,677,359	41,540	7,503	6092	262	1,654	4176	73.89%
Donor 2 Nuclei_droplet_PolyA	324,541,334	33,157	9,788	11589	581	1,309	9699	45.25%

Extended Table 2. Sequencing metrics. Note: "Total cells" refer to the number of cells classified by cellranger prior to any analysis, whereas "Cells post processing" refer to the number of cells remaining in our dataset post doublet and ambient RNA removal, mitochondrial and haemoglobin filtering and upper and lower UMI and gene thresholding.

Library	NK/T	B	Myeloid	Hofbauer	Plasma	HSC
Donor 1 FFPE_droplet_Probe (10X)	9	1	263	275	0	0
Donor 1 Fixed_droplet_Probe (10X)	3894	1837	1571	0	6	19
Donor 1 Fixed_in_situ_PolyA (Parse)	2569	1183	872	168	7	10
Donor 1 Fresh_droplet_PolyA (10X)	2042	1145	195	88	9	0
Donor 1 Nuclei_droplet_PolyA (10X)	138	47	0	1028	1	0
Donor 2 FFPE_droplet_Probe (10X)	267	3	389	615	272	2
Donor 2 Fixed_droplet_Probe (10X)	7885	487	1345	1	91	46
Donor 2 Fixed_in_situ_PolyA (Parse)	2015	105	168	366	347	22
Donor 2 Fresh_droplet_PolyA (10X)	2804	205	65	139	47	0
Donor 2 Nuclei_droplet_PolyA (10X)	248	53	0	1170	17	0

Library	STB	EVT	Fibroblast	Endothelial	CTB	Decidual	Myofibroblast
Donor 1 FFPE_droplet_Probe (10X)	1580	489	331	273	349	1	97
Donor 1 Fixed_droplet_Probe (10X)	1073	16	0	2	39	0	0
Donor 1 Fixed_in_situ_PolyA (Parse)	551	20	0	0	0	0	0
Donor 1 Fresh_droplet_PolyA (10X)	547	0	0	0	39	0	0
Donor 1 Nuclei_droplet_PolyA (10X)	4882	229	545	299	357	0	348
Donor 2 FFPE_droplet_Probe (10X)	1965	471	291	353	202	179	75
Donor 2 Fixed_droplet_Probe (10X)	1801	1	1	34	38	0	0
Donor 2 Fixed_in_situ_PolyA (Parse)	381	8	0	0	0	0	0
Donor 2 Fresh_droplet_PolyA (10X)	810	0	0	0	9	0	0
Donor 2 Nuclei_droplet_PolyA (10X)	5435	10	873	747	406	0	513

Extended Table 3. Cell numbers per cell type and modality.
